# Correlation of HTLV-1 proviral load and lymphocyte proliferation from asymptomatic HTLV-1-positive patients and HAM/TSP patients associated or not to skin disorders

**DOI:** 10.1186/1742-4690-11-S1-P16

**Published:** 2014-01-07

**Authors:** Renata Okajima, Jerusa Smid, Alberto JS Duarte, Augusto Cesar Penalva de Oliveira, Jorge Casseb, José Antonio Sanches Junior

**Affiliations:** 1 HTLV-outpatient Clinic, Institute of Infectious Diseases “Emilio Ribas”, São Paulo, Brazil; 2 Department of Dermatology, University of São Paulo Medical School, Brazil; 3 Department of Clinical Pathology, University of São Paulo Medical School, Brazil; 4 Institute of Tropical Medicine of São Paulo, University of São Paulo Medical School, Brazil

## 

This study evaluated the prevalence of skin diseases among HTLV-1 infected and the relation between HTLV-1 proviral load, and CD4+ and CD8+ T cells count among, regardless of clinical status, with or without associated skin disorders.193 HTLV-1-infected subjects were studied. Patients were submitted to a complete dermatological examination, lymphocyte proliferation assay (LPA), assay for HTLV-1 proviral load, CD4+ and CD8+ T cells count. A total of 147 patients had an abnormal skin condition; 116 (79%) of these patients also had skin disorder associated with HTLV-1 infection (SD-HTLV-1) (xerosis/ichthyosis or seborrheic dermatitis. The most prevalent SD-HTLV-1 was xerosis/acquired ichthyosis (49%), followed by seborrheic dermatitis (27%). Three of them had the association of adult onset IDH and HAM/TSP. The patients with SD-HTLV-1 were older (51 vs. 47 years), had a higher prevalence of myelopathy/tropical spastic paraparesis (HAM/TSP) (p=0.015), higher HTLV-1 proviral load (p=0.009) and had an increased 3-day basal LPA compared with patients without SD-HTLV-1 (p=0.008). T CD4+ and CD8+ cells counts show no significance. When HAM/TSP patients were excluded from the analysis, the HTLV-1 proviral load showed a significant difference (p=0.021), while LPA showed no difference. There was a high prevalence of skin disorders (76%) among HTLV-1-infected individuals, regardless of clinical status Initial HTLV-1 proviral load and age was higher in SD-HTLV-1 individuals, but the LPA showed an increase only in SD-HTLV-1 subjects with HAM/TSP.

